# An Additional Collaborator

**Published:** 1901-04

**Authors:** 


### AN ADDITIONAL COLLABORATOR.

IT will be observed that Dr. William Leon Ellerbeck has been appointed a collaborator to this journal. His contributions will be reviews of papers upon chemical and metallurgical subjects pertinent to dentistry; with the ultimate intention to include those upon bacteriology.

THE INTERNATIONAL DENTAL PUBLISHING COMPANY.

